# An international comparison of deceased and living organ donation/transplant rates in opt-in and opt-out systems: a panel study

**DOI:** 10.1186/s12916-014-0131-4

**Published:** 2014-09-24

**Authors:** Lee Shepherd, Ronan E O’Carroll, Eamonn Ferguson

**Affiliations:** Department of Psychology, Northumbria University, Newcastle-upon-Tyne, NE1 8ST UK; Department of Psychology, University of Stirling, Stirling, FK9 4LA UK; School of Psychology, University of Nottingham, Nottingham, NG7 2RD UK

**Keywords:** Opt-in consent, Opt-out consent, Deceased organ donation, Living organ donation

## Abstract

**Background:**

Policy decisions about opt-in and opt-out consent for organ donation are based on limited evidence. To fill this gap we investigated the difference between deceased and living organ donation rates in opt-in and opt-out consent systems across a 13 year period. We controlled for extensive covariates and estimated the causal effect of consent with instrumental variables analysis.

**Method:**

This panel study used secondary data analysis to compare organ donor and transplant rates in 48 countries that had either opt-in or opt-out consent. Organ donation data were obtained over a 13-year period between 2000 and 2012. The main outcome measures were the number of donors, number of transplants per organ and total number (deceased plus living) of kidneys and livers transplanted. The role of consent on donor and transplant rates was assessed using multilevel modeling and the causal effect estimated with instrumental variables analysis.

**Results:**

Deceased donor rates (per-million population) were higher in opt-out (*M* = 14.24) than opt-in consent countries (*M* = 9.98; *Β* = −4.27, 95% confidence interval (CI) = −8.08, −0.45, *P* = .029). However, the number of living donors was higher in opt-in (*M* = 9.36) than opt-out countries (*M* = 5.49; *B* = 3.86, 95% CI = 1.16, 6.56, *P* = .006). Importantly, the total number of kidneys transplanted (deceased plus living) was higher in opt-out (*M* = 28.32) than opt-in countries (*M* = 22.43; *B* = −5.89, 95% CI = −11.60, −0.17, *P* = .044). Similarly, the total number of livers transplanted was higher in opt-out (*M* = 11.26) than opt-in countries (*M* = 7.53; *B* = −3.73, 95% CI = −7.47, 0.01, *P* = .051). Instrumental variables analysis suggested that the effect of opt-in versus opt-out consent on the difference between deceased and living donor rates is causal.

**Conclusions:**

While the number of deceased donors is higher than the number of living donors, opt-out consent leads to a relative increase in the total number of livers and kidneys transplanted.

## Background

With the aim to increase the number of organs for transplantation, national health authorities face the conundrum of whether they should change from an opt-in to an opt-out consent system or visa-versa, or stick with their current system. This is a key health policy question facing all health services worldwide. Indeed, within the UK, Wales has recently decided to change from opt-in to opt-out consent. This is an area where opinions are strong and evidence is weak, and there is little well-controlled scientific evidence on which to base policy decisions. The aim of this research is to address three key gaps in knowledge by examining the effect of opt-in versus opt-out legislation (1) on both the number of deceased and living donations, (2) on transplantation rates for different types of organs and (3) as a causal factor.

There are sound reasons to believe that deceased organ donation rates will be lower in opt-in than opt-out consent systems. First, opt-out consent systems are likely to bridge the gap between people’s intentions and their behavior by removing the need to undertake any actions in order to become an organ donor [1]. Second, people may believe that defaults are policy-makers’ recommended course of action and act in accordance with this [1,2]. As a result, people should be more inclined to donate their organs when the default is to be a donor (such as in opt-out countries) than when the default is not to donate one’s organs (such as in opt-in countries). Finally, people are likely to view failing to donate one’s organs as more significant in opt-out than opt-in countries [3]. In line with these arguments, research has found that donation rates for heart beating donors diagnosed as brain stem dead in intensive care (that is, donation after brainstem death or DBD donors) are higher in opt-out than opt-in consent countries [4-8] and that organ donor rates increase after the introduction of opt-out consent [9].

The above evidence suggests that the introduction of opt-out consent is likely to increase the number of organ donors. However, there are three major problems with this research. First, the majority of the studies focused specifically on DBD donor rates. Although an important index of organ donation, the effect of opt-out consent becomes less clear-cut once other forms of organ donation are considered, such as living organ donation. There are good reasons why the majority of existing research has focused on the effect of consent on the deceased rather than the living donor rate; mainly that deceased donors produce a greater number and variety of organs. However, given that the majority of people on transplant waiting lists require a kidney and that more than a third of the total kidneys donated in the UK between 2012 and 2013 came from living donors [10], it seems reasonable to suggest that research should assess the effect of consent on both types of donation. This issue is especially important given that living kidney transplants are greater in opt-in than opt-out countries [11]. Also while the focus of consent type policy is specifically targeted at deceased donations, it is not clear how, or even if, opt-in or opt-out policies influence the living donations rate epiphenomenally. That is, an intervention targeted at one behavior influences a second potentially related behavior that it is not the target for. Second, previous research has focused on the number of deceased donors regardless of the type of organ. It is unclear whether opt-out consent increases the number of transplants regardless of organ type. It is important to acknowledge that the number of transplants will be influenced by the number of donors, as well as other factors, such as quality of health care provided and availability of trained surgeons. Given that the majority of transplants are for kidneys [10], it is possible that the higher levels of donation in opt-out consent systems may be predominantly due to this specific organ and that there is little difference for other organs, such as hearts, lungs and livers. Although there is some research assessing the role of opt-out consent on specific types of organ transplants [6,11], to our knowledge no previous study has compared transplant rates for a variety of organs in a large number of opt-in and opt-out consent countries, over an extended period of time, while attempting to control for as many potential covariates as possible.

A third key problem with research in this area is that it is inevitably observational rather than experimental. As a result, causality cannot be inferred. Fabre and colleagues [12] argue that because Spain’s rise in organ donation rates occurred 10 years after the introduction of opt-out consent, such legislation is unlikely to play an immediate causal role. Spain’s rise in donation rates occurred after the introduction of what is now known as the ‘Spanish Model’. This involved creating a transplant coordination network that operated at different levels (hospital, regional and national level), placing transplant coordinators at each procurement hospital, and improving the quality of information received by the general public [13]. Researchers have argued that the positive impact of opt-out consent on deceased donor rates may be due to the introduction of this model rather than opt-out consent alone [12]. However, because an effect takes time to emerge does not mean it is not a causal factor that produced the intervening changes that led to the increase. It should be seen as part of a causal change rather than a single casual factor. Therefore, consent type may still play a causal role. In such situations where it is impractical to conduct experimental research, instrumental variable (IV) regression models are one method that may be used to estimate a causal relationship [14].

The aim of the present study was to address these limitations and extend previous research by assessing the effects of opt-out versus opt-in consent legislation on (1) the number of deceased and living donors per million people in the population (or pmp), (2) the number of deceased (kidneys, livers, hearts and lungs) and living (kidneys and livers) transplants that occur for each type of organ (pmp), and (3) whether a causal relationship could be estimated using IV regression. In line with previous research [1,5,11], we tested transplant rates and donor rates relative to the population size (that is, pmp) to avoid the number of people in the population biasing the estimates. The panel study reported in this paper investigated organ donation and transplant rates in 48 countries (23 opt-in and 25 opt-out) between the years 2000 to 2012. Moreover, we also obtained data on the following covariates to ensure that any effects of opt-out versus opt-in consent system on organ donation was not explained by the following variables: road traffic accident mortality rate, gross domestic product (GDP; per capita, US $), the number of hospital beds (per 10,000 population) and the percentage of the population that self-identified as Catholic. In the IV regression analyses the instruments used were the legal system (whether the country is more likely to use civil or common law) and the percentage of people in each country involved in non-health based philanthropy (for example, volunteering time to an organization, helping a stranger and donating money to a charity; for a justification of these instruments, see below).

## Methods

**Figure 1:**
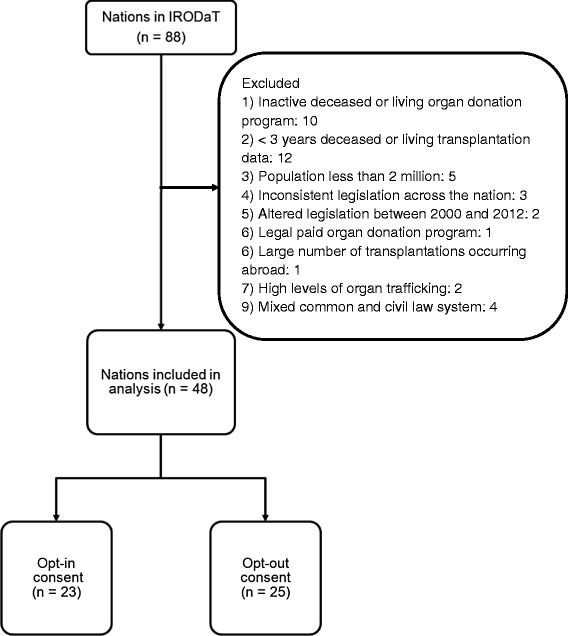
**Study flow diagram.**

To be included in the study a country must have published its organ donation and transplantation statistics on the Transplant Procurement Management’s International Registry of Organ Donation and Transplantation (IRODaT). This is an open and free database that is easily accessible for researchers. The data are provided by officials in each country, who are likely to be part of health ministries or members of national transplant organizations. At the time when the data were collected there were data available for 88 countries. A total of 48 of these countries met our inclusion criteria (23 opt-in and 25 opt-out) and were included in the sample (for details, see Figure 1 and Table 1). Complex longitudinal models usually require at least three data points [15]. Therefore, we only included countries with three or more years of deceased and living organ donor data between 2000 and 2012 to ensure that a reliable estimate was obtained. Countries were also excluded if they had a population below two million in 2000 because the reported statistics are based on donation per million population and countries with small populations would bias these data [5]. This is likely to occur through the creation of outliers and by inflating the mean donor and donation rate of the consent system under which these countries operate. Countries were also excluded if they had inconsistent organ donation legislation across the nation, had changed their consent system in the 13 year period under investigation, had paid organ donor programs, or high levels of organ transplants occurring abroad (that is, a high number of residents going abroad to receive a transplant [11]). Moreover, we also excluded countries that were reported to have high levels of organ trafficking and countries that had a mixture of civil and common law (see Table 2).

**Table 1:** **Countries included into the analyses**

**Country**	**Consent system**	**Source of consent information**
Argentina	Opt-out	4,11,17
Australia	Opt-in	4,5,11,16,17
Austria	Opt-out	4,5,11,16,17
Belarus	Opt-out	16
Belgium	Opt-out	4,5,11,16,17
Brazil	Opt-in^a^	4,16
Bulgaria	Opt-out	4,5
Canada	Opt-in	4,5,11,16,17
Columbia	Opt-out	11,16,17
Costa Rica	Opt-out	4,16
Croatia	Opt-out	4,5,11,16,17
Cuba	Opt-in	11,16
Czech Republic	Opt-out	4,5,11,16,17
Denmark	Opt-in	4,5,11,16,17
Ecuador	Opt-out	11,16
Finland	Opt-out	4,5,11,16
France	Opt-out	4,5,11,16,17
Germany	Opt-in	4,5,11,16
Greece	Opt-out	4,5,11
Guatemala	Opt-in	11
Hong Kong	Opt-in^b^	www.organdonation.gov.hk/eng/knowmore.html
Hungary	Opt-out	4,5,11,17
Republic of Ireland	Opt-in	4,5,11,16
Israel	Opt-in^c^	http://www.health.gov.il/English/Topics/organ_transplant/Pages/organs_donors.aspx
Italy	Opt-out	4,5,11,16,17
Japan	Opt-in	5,11,16
Latvia	Opt-out	4,5
Lebanon	Opt-in	17
Lithuania	Opt-in^d^	http://www.transplantacija.lt/content/apiemus.en.html
Malaysia	Opt-in	11,16,17
Mexico	Opt-in	11,16
The Netherlands	Opt-in	4,5,11,16,17
New Zealand	Opt-in	4,5,11,16,17
Panama	Opt-out	4,11
Poland	Opt-out	4,5,11,16,17
Portugal	Opt-out	4,5,11,17
Puerto Rico	Opt-in	18
Romania	Opt-in	4,5,16
Russia	Opt-out	16
Singapore	Opt-out	4,11,16
Slovak Republic	Opt-out	4,5,11,16,17
Spain	Opt-out	4,5,11,16
Sweden	Opt-out	4,5,11,16,17
Taiwan	Opt-in^b^	http://www.nhi.gov.tw/English/webdata/webdata.aspx?menu=11&menu_id=594&WD_ID=594&webdata_id=3172
Tunisia	Opt-out	11,16
UK	Opt-in	4,5,11,16,17
USA	Opt-in	4,5,11,16,17
Venezuela	Opt-in	4,11,16

^a^Brazil’s opt-out law was abolished in 1998 [16]. Therefore, this was regarded as an opt-in country despite the fact that the opt-in legislation was not in in place until 2001 [4]. ^b^According to the source people are required to join a register if they wished to donate their organs. Therefore, this was regarded as an opt-in country. ^c^Israel’s consent legislation has been categorized as opt-in by some studies [17,18] and opt-out by others [4,5]. According to the source people are required to sign a donor card to testify that they wish to donate their organs after their death. Therefore, we regarded Israel as an opt-in country. ^d^The majority of research [4,5,11,17] regards Lithuania as an opt-in country. However, one study [18] regarded this country as opt-out. In this country people can register their consent and dissent for organ donation. If people have not registered this, then consent is requested from next-of-kin. If the next-of-kin cannot be contacted, donation can only occur if it is an emergency. In all other cases consent is required from either the deceased or their next-of-kin. Therefore, we concurred with the vast majority of previous research in categorizing Lithuania as an opt-in country.

**Table 2:** **Countries excluded from the analyses**

**Reason**	**Countries excluded for this reason**
Inactive deceased or living donor program^a^	Armenia, Azerbaijan, Bangladesh, Egypt, El Salvador, Georgia, India, Libya, Luxembourg, Macedonia
Less than three years of living or deceased data^b^	Bahrain, Bolivia, Brunei, Jordan, Kuwait, Morocco, Nicaragua, Pakistan, Paraguay, Peru, Slovenia, Trinidad and Tobago
Population less than two million^c^	Cyprus, Estonia, Iceland, Malta, Qatar
Inconsistent consent legislation^d^	Dominican Republic, Switzerland, Turkey
Changed legislation in 13 year period^e^	Chile, Uruguay
Legal paid system^d^	Iran
Large number of transplants occurring abroad^d^	Saudi Arabia
Reports of high levels of organ trafficking^f^	Moldova, Ukraine
Mixed civil and common laws^g^	Norway, Philippines, South Africa, South Korea

^a^Data from the IRODaT database indicated that the country had not performed the transplant in the 13 year period under investigation. ^b^Based on data from the IRODaT database. ^c^Based on population statistics from the U.S. Census Bureau. This was based on the population for 2000 because this was the first year under investigation. ^d^Based on previous research [11]. ^e^Based on previous research [11,17]. ^f^This was based on news reports [19,20]. ^g^Based on data from the World Factbook. Although parts of the USA (Louisiana) and Canada (Quebec) use civil law, these were regarded as common law countries because this is the dominant legal system. Similarly, although Spain has regional variations in the legal system it predominantly uses civil law and was, therefore, categorized as such. Although Japan’s legal system is influenced by Anglo-American law, it is based on the German model of civil law and was, therefore, regarded as a civil law country.

### Data sources

The number of deceased and living donors, as well as the number of transplants per organ, were obtained from the IRODaT database. The deceased organ donor data consisted of both DBD and donation after cardiac death (DCD) donors (if applicable). In line with IRODaT, any donor or transplant score with a value of zero was regarded as missing data. These data did not differentiate between adult and child donors. Each country’s organ donation consent legislation (scored −1 for opt-in and +1 for opt-out) was obtained from previous research [4,5,11,17,18,21]. There were some countries that were either not included in this research or were categorized as having opt-in consent in some studies and opt-out consent in other studies. For these countries, legislation data was obtained from websites belonging to the government or professional organizations (see Table 1). In line with previous research [1,4,5,7,11], GDP, whether the legal system was more influenced by common or civil laws (scored −1 for common law and +1 for civil law), the percentage of self-identified Catholics (scored −1 for ≤25%, 0 for >25% to 75%, and 1 for >75%), number of hospital beds (per 10,000 population), and road traffic accident (RTA) fatality rate pmp were entered into the analysis as covariates. GDP was entered into the analysis because this variable is positively associated with deceased organ donation rates [11]. Previous research has found that opt-out countries are likely to be predominantly Catholic [5]. Moreover, deceased donor rates are greater in countries with a high proportion of Catholics [8]. Therefore, in line with previous research [5,11], it was important to control for this variable. The number of hospital beds was included in the model as an estimate of the quality of the healthcare infrastructure in each country. This ensured that any effect of consent was not due to opt-out countries having a high-quality healthcare infrastructure. Finally, countries with higher levels of RTA mortalities may be more likely to have a large supply of donor organs [5,7,11]. The inclusion of these covariates ensured that any effect of consent legislation on organ donation was not in fact due to these factors. We obtained population data from the US Census Bureau in order to calculate the pmp estimates. Finally, the type of legal system was entered into the analysis because this variable is associated with the consent system and, as such, was also examined as an IV [5]. The sources of all the data are presented in Table 3.

**Table 3:** **Sources of the data for the study**

**Variable**	**Source**	**Website**	**Permission needed**
Organ donation data	International Registry of Organ Donation and Transplantation*	http://www.irodat.org/ (accessed 19/08/2013)	No: http://www.irodat.org/?p=about
GDP per capita (US$)	International Monetary Fund (2013), *World Economic Outlook database,* Data and Statistics IMF*	http://www.imf.org/external/pubs/ft/weo/2013/01/weodata/download.aspx (accessed 12/08/2013)	Yes: Permission Sought and Attained
	The World Bank: GDP per capita: World Development Indicators	http://data.worldbank.org (accessed 12/08/2013)	No:http://web.worldbank.org/WBSITE/EXTERNAL/0,,contentMDK:22547097~pagePK:50016803~piPK:50016805~theSitePK:13,00.html
Legal system	Central Intelligence Agency World Factbook*	https://www.cia.gov/library/publications/the-world-factbook/fields/2100.html#133 (accessed 19/08/2013)	No:https://www.cia.gov/library/publications/the-world-factbook/docs/contributor_copyright.html
Catholicism	Central Intelligence Agency World Factbook*	https://www.cia.gov/library/publications/the-world-factbook/fields/2122.html#195 (accessed 12/08/2013)	No: see above link for Legal System
	U.S. Department of State	http://www.state.gov/j/drl/rls/irf/2010/index.htm (accessed 12/08/2013)	No:http://www.state.gov/misc/87529.htm#copyright
RTA mortality rate	*Global status report on road safety: time for action*. Geneva, World Health Organization, 2009*	http://www.who.int/violence_injury_prevention/road_safety_status/2009 (accessed 12/08/2013)	Yes: Permission Sought and Attained
	Based on data from ‘OECD (2010), *IRTAD Road Safety Annual Report 2009*, OECD Publishing’	http://dx.doi.org/10.1787/9789282102824-en (accessed 12/08/2013)	Yes: Permission Sought and Attained
	Hong Kong Road Safety Council	http://www.roadsafety.gov.hk/annual_report/2006/eng/foreword.html (accessed 12/08/2013)	Yes: Permission Sought and Attained
	Taiwan’s Ministry of Transportations and Communications	http://www.motc.gov.tw/en/home.jsp?id=255&parentpath=0,150,250 (accessed 12/08/2013)	Yes: Permission Sought and Attained
Population	U.S. Census Bureau*	www.census.gov/population/international/data/idb/informationGateway.php (accessed 13/08/2013)	Yes: Permission Sought and Attained
Helping a stranger	Charities Aid Foundation (2010, 2011, 2012), World Giving Index*	https://www.cafonline.org/pdf/WorldGivingIndex28092010Print.pdf (accessed 13/08/2013)	Yes: Permission Sought and Attained
	This is based on data from Gallup’s WorldView World Poll		
		https://www.cafonline.org/pdf/World_Giving_Index_2011_191211.pdf (accessed 13/08/2013)	Yes: see above information for World Giving Index 2010
		https://www.cafonline.org/PDF/WorldGivingIndex2012WEB.pdf (accessed 13/08/2013)	Yes: see above information for World Giving Index 2010
Hospital beds	*World Health Report: health systems: essential health technologies.* Geneva, World Health Organization, 2013*	http://apps.who.int/gho/data/node.main.70?lang=en (accessed 30/09/2013)	Yes: Permission Sought and Attained
	National Statistics Republic of China (Taiwan)	http://eng.stat.gov.tw/lp.asp?ctNode=2267&CtUnit=1072&BaseDSD=36&MP=5 (accessed 30/09/2013)	Yes: Permission Sought and Attained
	Information Services Department, Hong Kong Special Administrative Region Government	http://www.gov.hk/en/about/abouthk/factsheets/docs/public_health.pdf (accessed 30/09/2013)	No:http://www.gov.hk/en/about/abouthk/factsheets/docs/public_health.pdf
	The World Bank: Hospital beds (per 1,000 people): World Health Organization	http://data.worldbank.org (accessed 30/09/2013)	No: see above link for GDP per capita for The World Bank

* = Primary source. For Cuba the number of self-identified Catholics was prior to Castro assuming power. The data for 1996 was used to estimate Puerto Rico’s hospital beds because this was the most reliable information that we were able to obtain. GDP, gross domestic product; RTA, road traffic accidents.

### Statistical analysis

Organ donation and transplant rates across the 13-year period (2000 to 2012) were nested within countries. As such, multi-level modeling (MLM) is the appropriate statistical technique to assess the effect of country level variables (for example, consent) on the within country variation in donation rates. If this effect of nesting is not accounted for in the statistical model the standard errors (and, hence, significance) will be distorted by conflating variation at one level (donation rate over time) with another (country). Thus, the use of MLM provides a more accurate overall assessment of the effect of consent (which varies across country) on donation rate (which varies within country). In each analysis we excluded countries that had not transplanted the organ in question over the 13-year period because this indicated an inability or reluctance to transplant this organ. Consent system (opt-in versus opt-out) was entered into the model as a factor. Legal system, GDP, RTA pmp, hospital beds, and the percentage of Catholics were entered as covariates as between countries (Level 2 variables). These covariates were all time invariant. The mean GDP over the 13-year period was used in the analysis.^1^ Years (2000 to 2012) were coded 1 to 13 and were a repeated measure (Level 1) factor. Organ donation/transplantation rates per year were the outcome variables. The continuous Level 2 variables (GDP, RTA and hospital beds) were grand mean centered. The intercept was based on the mean level GDP, RTA and hospital beds, and the proportion of countries in each of the legal and Catholicism categories. The initial models were random intercept models with year specified as a random slope. These analyses were repeated for both deceased and living donor rates, and for the transplantation rates for each organ. These MLM analyses were conducted in SPSS (Version 21). The multi-level path model was specified in M*plus* 7 [22].

The IV regression approach attempts to untangle problems such as reverse causation (that is, whether consent affects donation rates or visa-versa) and missing variables in the model. IV regression estimates the causal relationship between the endogenous predictor (consent), by identifying IVs (correlated with the predictor, unrelated to the outcome and orthogonal to the errors). As the instrumental variable is associated with the predictor (consent) and not the outcome (or the error term) it breaks the predictor into the part associated with error and the part that is not. By isolating the part of the predictor that is not associated with error it is possible to infer causal links between the predictor and outcome [14]. IV regression requires large sample sizes [14,23]. In this area of research large sample sizes based on between country comparisons alone are unlikely to be achieved. One way around this problem is to take advantage of the panel data structure and apply Baltagi’s [24] error component two-stage least squares (EC2SLS) approach to estimate IV regression in panel data. This approach was implemented in Stata 13.

Two classes of IV were identified: legal system (common or civil law) and levels of non-health related philanthropy in each country. Civil law systems, compared to common law, are generally more prescriptive. Legislation for public goods is, therefore, more likely, and as such they should be more likely to adopt an opt-out consent system [5]. However, the variation in legal systems should not directly affect the supply of organs (living or deceased), only via consent.

Countries that have higher norms for non-health related philanthropy may also prefer an opt-in system of consent. Higher levels of non-health related philanthropy are likely to be associated with a more active attitude towards helping and giving. Indeed, people in opt-in countries are more likely to see the act of organ donation as a meaningful and active process, perhaps reflecting a general norm that giving is an active process [3]. Thus, we expect that countries with an opt-in policy will exhibit higher levels of non-health related philanthropy (helping strangers, volunteering and donating money). That is, where the countries attitude towards non-health related philanthropy is positive, this will reflect giving as an active process, and in such countries the more active opt-in consent process will be favored. This higher non-health philanthropy in opt-in countries should influence donation via the consent process only. In support of this contention there is evidence to suggest that health based philanthropy (for example, blood and potentially organ donation) is not related to non-health based philanthropy [25-27]. However, while both deceased and living donation rates may be viewed as altruistic, living donation is a more definitive altruistic act – it is at a cost to the donor, voluntary, and a benefit to the recipient (there is no cost to the donor for deceased donations) [28]. To avoid this potential problem for applying IV regression we examine the potential causal role of consent on the difference between living and deceased donation rates in each country by year. This also allows us to control in the models for any association between living and deceased donation rates that may be related in a compensatory manner (high deceased donation rates linked to lower living rates and visa-versa) within countries. Thus non-health based philanthropy should be associated with the consent system but not the difference in living versus deceased organ donor rates. Non-health related philanthropy was estimated by the percentage of people in each country who were willing to help a stranger, volunteer or donate money. These data were obtained from the World Giving Index (WGI) for the years 2010, 2011 and 2012 and the average entered in the model for all 13 years (see Table 3).

### Ethics

All the data used in this reported panel study are publically available data (all sources and links to the original data are provided) and the study was approved by the Faculty of Health and Life Sciences Ethics Committee of Northumbria University (reference RE-HLS-12-130704-51d53de10a88b) on the 8 July 2013. Where required we requested and obtained permission to use the data sources reported in this paper (see Table 3, final column).

## Results

### National data

There were 48 countries in the final data set. For the total deceased donors the number of years of data ran from 3 to 13 years with a mean of 10.85 years (SD = 2.94). For the living donors the total number of years ran from 3 to 13 years with a mean of 9.56 years (SD = 2.98). The number of countries did not systematically vary as a function of self-identified Catholic bands (≤25%, >25% to 75%, and >75%: *χ*^*2*^ (2) = 3.88, *P* = .144). There were significantly more civil (N = 38, 79%) than common law countries (N = 10, 21%; *χ*^*2*^ (1) = 16.33, *P* < .001). The association of consent with the national variables is presented in Table 4. The only significant effect was an association between the consent system and the legal system, with common law being more likely in opt-in than opt-out consent countries.

**Table 4:** **Association of opt-out legislation to national variables**

**Predictors**		**Opt-in** ***M*** **(SD)**	**Opt-out** ***M*** **(SD)**	**Statistic**
GDP		23,313.06 (15,712.19)	17,229.90 (13,534.59)	*F*(1, 46) = 2.08, *P* = .157, η_p_^2^ = .04
RTA		108.60 (62.45)	120.16 (41.95)	*F*(1, 46) = 0.58, *P* = .452, η_p_^2^ = .01
Hospital beds		44.56 (27.77)	50.98 (26.57)	*F*(1, 46) = 0.67, *P* = .417, η_p_^2^ = .01
		Opt-in	Opt-out	
		N	N	
Catholic	≤25%	11	10	*χ* ^*2*^(2) = 1.84, *P* = .399
	>25% to 75%	6	4	
	>75%	6	11	
Law	Civil	14	24	*χ* ^*2*^(1) = 8.96, *P* = .003*
	Common	9	1	

*This estimate is based on Pearson Chi-Squared. Given the low number of opt-out and common law countries the Yate’s continuity correction (*χ* ^*2*^(1) = 6.96, *P* = .008) or Fisher’s Exact Test (*P* = .004, two-tailed) were applied. Both were significant. GDP, gross domestic product; RTA, road traffic accidents pmp.

### Organ donor and transplant rates

The intra-class correlations were .89 for deceased donation and .85 for living donation. This indicates that 89% of the variation in deceased donation rates is attributable to variation at the country level, as is 85% of the variation in living donation rates. This indicates that MLM is the appropriate analytic strategy for these data. As such, we initially ran two separate random intercepts MLMs with year specified as a random slope, comparing the effect of (opt-in (N = 23) versus opt-out (N = 25) consent) and the covariates on the number of either deceased or living donors. The estimated effect of opt-in versus opt-out consent was based at the average GDP, RTA, hospital beds and with Catholicism and legal system relative to the proportion in each category. The results show that across the dataset, there were significantly more deceased donors in opt-out than opt-in consent systems (Table 5). However, there were significantly more living donors in opt-in than opt-out consent systems. This effect remains after controlling for the covariates, indicating that consent had a unique effect on both deceased and living donation rates. Importantly, the number of living and deceased donors increases over the years. We reanalyzed these data with Spain removed from the analyses. We removed Spain because it is a well-known system with a strong opt-out policy that may influence the results. Thus, to test that the effects were not due to unique factors associated with the Spanish system we re-ran the models excluding Spain [5]. The pattern of results was the same once Spain was removed (Table 5), indicating that the findings were not due to anything unique about the Spanish model. Moreover, we also reanalyzed the data to examine the cross-level interaction of consent with years on both deceased and living donor rates. This interaction was not significant for either deceased (*P* = .28) or living donors (*P* = .46). Thus, the effect of consent was constant across the years.

**Table 5:** **The impact of opt-out consent on organ donation rates (pmp), 2000–2012**

**Type of donation**	**Overall grand mean**	**Opt-in consent**	**Opt-out consent**							
	**(No. countries, total observations)**	***M*** **(** ***SE*** **)**	***M*** **(** ***SE*** **)**	**Consent**	**Year (** ***B*** **)**	**GDP (** ***B*** **)**	**RTA (** ***B*** **)**	**Catholic (** ***B*** **)**	**Legal (** ***B*** **)**	**Hospital beds (** ***B*** **)**
Deceased donors	12.11 (48, 521)	9.98 (1.30)	14.24 (1.28)	*t*(41.24) = 2.26, *P =* .029	0.29***	0.0004***	0.01	3.19**	0.28	0.01
Living donors	7.42 (48, 459)	9.36 (0.95)	5.49 (0.94)	*t*(41.11) = 2.89, *P =* .006	0.26**	0.0001*	−0.002	−2.05*	1.11	−0.09**
Without Spain										
Deceased donors	11.64 (47, 508)	9.87 (1.19)	13.41 (1.19)	*t*(39.99) = 2.06, *P* = .046	0.29***	0.0004***	0.01	2.59*	0.07	0.02
Living donors	7.47 (47, 448)	9.33 (0.95)	5.60 (0.97)	*t*(40.14) = 2.75, *P =* .009	0.26**	0.0001*	−0.002	−1.97*	1.13	−0.09**

Table contains estimated means and standard errors. *P < .05, **P < .01, and ***P < .001. Consent scored −1 = opt-in, 1 = opt-out, Legal scored −1 = common law, 1 = civil law, Catholic scored −1 = ≤25%, 0 = > 25% to75%, 1 = >75%, continuous level-two variables (GDP, RTA and hospital beds) were grand mean centered. Coefficients are all unstandardized. GDP, gross domestic product; RTA, road traffic accidents.

**Figure 2:**
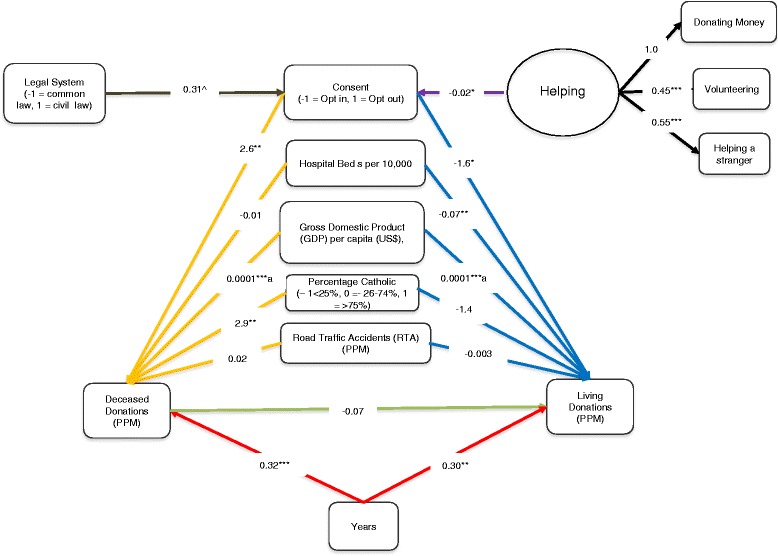
**Multi-level path model for prediction of deceased and living donation rates.** Legend. ^*P* = .089, *P < .05, **P < .01, ***P < .001. RTA, GDP and Hospital Beds are Grand Mean Centered. Slope between years and deceased and living donation rates and deceased and living donation rates are random. N = 450, with 47 clusters (countries). There was no helping data from Cuba. Therefore, this country was not included in the analysis, reducing the sample to 47 countries. Coefficients are unstandardized and the estimator is Maximum Likelihood with Robust Standard Errors. Blue paths represent the effect of covariates and consent on living donation rate, yellow paths represent the effect of covariates and consent on deceased donation rate, red paths represent the effect of years on both living and deceased donation rate, the green path is the effect of deceased donation rate on living donation rate. The purple path is the effect of the helping latent factor on consent and the brown path the effect of the legal system on consent. The black paths are the unstandardized factor loadings. ^a^Rounded as M*Plus* only reports to three decimal places. GDP, gross domestic product; RTA, road traffic accidents.

We also ran a multi-level path model to explore the interplay between the main study variables in more detail. In this model, we specified a random slope between deceased and living donation to examine if the deceased donation rate predicts the living donation rate. We also specified random slopes between years and donation rates (living and deceased). Deceased and living donation rates were treated as random intercepts predicted by the Level 2 covariates (GDP, RTA and hospital beds were grand mean centered). While this is a multi-level path model and not an IV regression model, we include the instruments (legal system and a latent factor representing non-health related philanthropy). This model is shown in Figure 2. There are two things of note. First, there is no significant association between deceased and living donation rates, and both are independently predicted by consent. Living donation rates are higher under opt-in and deceased under opt-out. The second point of note is that the potential instruments operate in the predicted manner. Greater non-health related philanthropy is associated with opt-in consent systems and civil law associated with opt-out consent.

When comparing the transplant rates for each type of organ we found that deceased kidney and liver transplants were higher in opt-out than opt-in consent systems (Table 6). There was a trend for deceased heart transplants to be higher in opt-out than opt-in countries, but this difference was not significant. The total number of deceased lung transplants did not differ between opt-out and opt-in consent systems. By contrast, there were significantly more living kidney transplants in opt-in than opt-out consent systems (*P* = .049). There was not a significant difference between the number of living liver transplants between opt-in and opt-out countries (*P* = .590). Importantly, the total number of kidney transplants (deceased plus living) was higher in opt-out than opt-in countries (*P* = .044). Similarly, the total number of liver transplants was higher in opt-out than opt-in countries (*P* = .051). There are also effects showing that organ donation rates are increasing over the years (both deceased and living) except for heart and lungs from deceased transplant and liver from living transplant.

**Table 6:** **The impact of opt-out consent on organ transplant rates (pmp), 2000–2012**

		**Grand mean (N**_**T**_ **, observed)**	**Opt-in consent**	**Opt-out consent**							
**Organs & Source**			***M*** **(** ***SE*** **) N**_**I**_	***M*** **(** ***SE*** **) N**_**o**_	**Consent**	**Years (B)**	**GDP (B)**	**RTA (B)**	**Catholic (B)**	**Legal (B)**	**Hospital beds (B)**
Deceased	Kidney	18.67 (48, 522)	14.27 (1.84) 23	23.07 (1.80) 25	*t*(40.88) = 3.29, *P* = .002	0.44***	0.0006***	0.02	4.08*	0.60	0.02
	Liver	7.19 (47, 470)	5.51 (0.96) 22	8.88 (0.95) 25	*t*(40.23) = 2.37, *P =* .022	0.23***	0.0003***	0.004	1.88*	−0.19	−0.02
	Heart	2.86 (45, 473)	2.40 (0.39) 21	3.32 (0.37) 24	*t*(38.18) = 1.63, *P =* .111	0.04	0.0001***	0.007	0.50	0.21	0.001
	Lungs	1.01 (31, 233)	0.79 (0.28) 17	1.22 (0.31) 14	*t*(23.37) = 0.97, *P =* .343	0.01	< 0.0001	−0.005	−0.37	0.05	−0.01
Living	Kidney	6.57 (48, 507)	8.01 (0.99) 23	5.13 (0.98) 25	*t*(40.80) = 2.03, *P =* .049	0.23**	0.0001*	0.01	−1.93*	0.93	−0.10***
	Liver	1.14 (39, 320)	1.27 (0.37) 20	1.02 (0.39) 19	*t*(34.66) = 0.54, *P =* .590	0.03	< −0.0001	−0.01^†^	0.22	−0.37	0.01
Total	Kidney	25.37 (48, 499)	22.43 (1.96) 23	28.32 (1.93) 25	*t*(40.91) = 2.08, *P* = .044	0.68***	0.0008***	0.02	2.29	1.06	−0.08
	Liver	9.39 (37, 309)	7.53 (1.20) 19	11.26 (1.28) 18	*t*(30.81) = 2.03, *P* = .051	0.32***	0.0003**	−0.003	1.60	−0.05	−0.02

^a^The Slovak Republic had data for deceased and living liver transplants. However, there were no deceased and living liver data for the same year. Therefore, we were unable to calculate total liver transplants for this country and it was removed from this analysis. ^†^ *P* = .085, *P < .05, **P < .01, and ***P < .001. N_T_ = total number of countries in analysis, observed = number of observations, N_I_ = number of opt-in countries in the analysis, N_o_ = number of opt-out countries in the analysis. Consent scored −1 = opt-in, 1 = opt-out, Legal scored −1 = common law, 1 = civil law, Catholic scored – 1 = ≤25%, 0 = > 25% to75%, 1 = >75%, continuous level-two variables (GDP, RTA and hospital beds) were grand mean centered. Table contains estimated means and standard errors. GDP, gross domestic product; RTA, road traffic accidents.

### Instrumental variables regression: predicting the difference in deceased and living donation rates

Separate random effects panel regressions with robust standard errors showed that the averaged donation of money in a county was not related to the difference in deceased and living donations (B = −0.04, *P* = .52), nor was volunteering (B = −0.03, *P* = .80), helping a stranger (B = −0.10, *P* = .32) nor the type of legal system (B = 2.2, *P* = .09). The first stage statistics from the panel IV regressions showed that of the four instruments, volunteering was not significantly associated with consent type (*P* = .12), the other three were (all *P*s < .001). Thus, volunteering was removed as an instrument. The first stage statistics for the final model with three instruments (legal system, the averaged donation of money and helping a stranger) showed that the type of legal system was significantly and positively associated with the type of consent (B = 0.03, Z = 3.4, *P* = .001), such that countries with a civil legal system were more likely to have an opt-out system. Also, donating money (B = −0.002, Z = −2.99, *P* = .003) and helping a stranger (B = −0.003, Z = −4.24, *P* < .0001) were significantly negatively associated with consent, such that levels of these types of non-health related philanthropy were higher opt-in countries.

Table 7 shows the results of the IV panel regression model. The first column is a Generalized Least Squares (GLS) analysis, with robust standard errors that replicates the majority of the main findings in Table 5 but for the difference score. The IV regression model shows that consent type predicts the relative prevalence of deceased versus living donations, such that opt-out consent results in proportionally higher levels of deceased donations. The Sargan-Hansen test was calculated using the xtoverid command of Schaffer and Stillman [29]. The Sargan-Hansen test indicates that the orthogonality constraint was met.

**Table 7:** **Results of instrumental variable regression analysis (EC2SLS) predicting the difference in deceased versus living donation rates**

**Predictor**	**GLS**	**EC2SLS**
Consent (−1 = opt-in, 1 = opt-out)	4.03**	4.09*
Years	0.11	0.11*
% Catholic	5.7***	5.3***
RTA	0.02	0.03
GDP	0.0003***	0.0003**
Hospital beds	0.10^^	0.09^
Overall R^2^	0.44	0.45
Sargan-Hansen test		3.82 (4) *P* = .43
Number of groups	48	47
Number of observations	457	450
Average cluster size (range)	9.5 (3, 13)	9.6 (3, 13)

^^*P* = .076, ^*P* = .052, *P < .05, **P < .01, ***P = .001. There are 47 countries in these analyses as there were no data on helping for Cuba. GDP, RTA and Hospital beds are Grand Mean centered. The outcome is the difference between deceased and living (deceased – living) donation rates, such that positive scores favor deceased donation and negative scores living conation. Unstandardized coefficients. EC2SLS, Error Component Two-Stage Least Squares; GDP, gross domestic product; GLS, Generalized Least Squares; RTA, road traffic accidents.

## Discussion and Conclusions

In terms of the policy dilemma posed at the start of this paper, the results show that opt-out consent may lead to an increase in deceased donation but a reduction in living donation rates. Opt-out consent is also associated with an increase in the total number of livers and kidneys transplanted.

Importantly, the relationship between deceased and living donor rates was non-significant, implying that on average, one is not compensating for the other. Indeed, this would be unlikely as the range of organs available from deceased donation is greater than that from living donation (only kidneys and liver lobes). We also found that the number of deceased and living donors, and the number of deceased kidney and liver transplants, has increased over the years from 2000 to 2012. This increase in donation and transplantation rates is likely to be due to a variety of factors, including not only an increase in the number of people willing to donate, but also improved criteria for identifying and selecting donors, improved transplantation procedures and an increase in transplantation capacity (that is, greater availability of surgeons, more transplant centers).

Unlike living kidney transplantation, living liver transplantations were not significantly greater in opt-in than opt-out countries. The mortality rate is significantly higher for living liver donation than for living kidney donation [30,31], which may make people more reluctant to use this alternative to deceased liver donation. If the number of donors is reduced so, as a consequence, is the number of potential transplants. Indeed, research suggests that patients may be reluctant to ask loved ones to donate part of their liver because of the potential guilt that they would feel if their living donor family member were to die during the procedure [32]. Moreover, there may also be a lack of trained surgeons to undertake this procedure, further reducing living liver transplant rates. There was also no significant difference between the number of lung transplants between opt-in and opt-out systems. While null results are hard to interpret, the lack of any systematic effect may reflect the low base rate in the availability of lungs for donation. There is a high eligibility criteria for lung transplants [33], which may reduce the likelihood of obtaining a deceased lung donor. Moreover, there is a higher mortality rate for lung transplants than kidneys and livers [34], reducing the likelihood of this procedure being undertaken. As a result, the number of deceased lung transplants in opt-in and opt-out consent countries is likely to be low.

The use of IV analysis enhanced previous research in this area by estimating the causal effect of consent on the difference in donation rates between deceased and living donors. This analysis revealed that consent was likely to influence the difference in donation rates between deceased and living donors organ donation, such that opt-out consent results in relatively greater deceased than living donations. These analyses, in combination with previous experimental research, further support a causal interpretation. For example, experimental vignette-based research has found that people were more willing to donate their organs when opt-out rather than opt-in legislation was used [1]. This experimental research demonstrates the causal effect of consent type on people’s support for organ donation.

### Factors influencing organ donation and transplantation

Although we support previous research in demonstrating greater deceased donor rates in opt-out than opt-in countries, it may be too simplistic to state that the introduction of opt-out consent will increase deceased donation rates. Indeed, there are examples where opt-out consent has not improved donor rates. For example, in France and Brazil the introduction of opt-out consent had a detrimental effect on donation, which was partly attributed to increased levels of mistrust towards medical professionals [16,35]. This possibility was one concern that led the Organ Donation Taskforce to conclude that opt-out consent should not be introduced to the UK in 2008. Although these case studies are informative, they do not constitute rigorous and scientific evaluation of the effect of consent on medical mistrust. Therefore, further empirical evidence is needed to determine whether levels of medical mistrust vary between opt-in and opt-out countries and to establish the effect of this on donation rates.

From the point of view of the results in this study, there are numerous reasons why it is unlikely that factors associated with the ‘Spanish Model’ can explain the results of the present study (indeed, our results remain the same when Spain is removed from the analyses). First, the factors in the Spanish Model (for example, multi-level transplant coordination network, hospital coordinators) cannot explain why living donation was lower in opt-out than opt-in countries. Second, the number of intensive care beds is often regarded as influencing the availability of organs [36]. We included the number of hospital beds in our model, as a general index of the quality of the healthcare infrastructure, and the effect of consent remained significant. However, it should also be noted that although the number of intensive care beds (per 100,000 people in the population) is higher in Spain than the UK, it is substantially lower than a number of opt-in countries, such as Germany, US, and Canada [37,38]. Indeed, the number of intensive care beds in Germany is more than twice as many as in Spain [37,38]. Moreover, there is not a significant difference between the number of critical care beds in opt-in and opt-out countries.^2^ In addition, the fact that aspects of the Spanish model have been introduced to both opt-in (for example, UK) and opt-out countries (for example, Italy) suggests that the differences found in the present study are unlikely to be due to the Spanish model.

While the Spanish model itself may not be able to explain the effect of consent, aspects of the Spanish Model are likely to be highly beneficial to deceased donor rates [39]. Indeed, one recommendation of the UK Organ Donation Taskforce [40] was to apply some aspects of the Spanish Model to the UK organ donation system. For example, in line with the Spanish Model clinical leads for organ donation have been appointed in each Hospital Trust and aim to liaise with the transplant team and the Hospital Trust to promote organ donation. Set against a general background of the number of transplants and donations increasing from 2000 to 2012, there has been a 50% increase in deceased donors since the publication of this report, which has been partly attributed to the implementation of such recommendations [41]. Importantly, this rise occurred without any change to the UK’s consent legislation. This clearly demonstrates the success of applying some aspects of the Spanish Model.

### Future research and implications

A limitation of this research is that it cannot account for the variability in the application of opt-out legislation. Some countries apply either ‘soft’ or ‘hard’ opt-out consent legislation. In soft opt-out consent countries donation cannot take place without the permission of family members. By contrast, in hard opt-out consent countries organs can be transplanted from anyone who has not registered their opposition to donation, regardless of whether or not their family members have been consulted. In the majority of opt-out consent countries the permission of next-of-kin is required when the deceased’s wishes are not known and next-of-kin can veto donation [17]. Therefore, the majority of these countries use soft opt-out consent. However, our results demonstrate a difference between opt-in versus opt-out consent countries despite this variability in the implementation of this legislation across different nations. Therefore, we found that overall opt-out consent is associated with greater deceased donor rates. Given the lack of data on the type of opt-out consent used in each country and the limited number of countries available for the analyses, it was not feasible to test for these differences using the current methodology. It remains for further research when enough data are available to make meaningful distinctions between opt-in versus both soft and hard opt-out systems.

It is also important to assess other factors that are likely to influence the organ donation system. For example, organ donation and transplantations are likely to be influenced by the use of the Spanish Model, the role of the organ procurement organizations and the capacity of the transplant system (for example, number of trained surgeons and transplant centers). Again, data availability may prevent researchers from assessing this using the current methodology. Thus, it is imperative for transplant organizations to routinely collect data on important organ donation indices (for example, consent type, procurement procedure, number of intensive care beds and trained surgeons) and make this publicly available to develop future research and policy recommendations in this area. Although such country-level data are informative, there are some limitations. For example, due to data availability it is not possible to infer the role of attitudes on consent type and donation rates. Therefore, future research should apply other methodologies to further test the effect of consent type. For example, researchers could use vignette-based experimental studies [1], other experimental laboratory-based work, such as economic games [42], or pre-post time series designs. By combining the findings from these different research methods, researchers could have a greater understanding of the factors that promote organ donation and transplantation.

It should also be noted that opt-out consent countries still have significant transplant waiting lists and suffer from an organ donor shortage. The introduction of opt-out consent legislation is, therefore, unlikely to totally solve a country’s organ shortage. Indeed, organ donation rates are multi-causal and a variety of factors need to be considered to improve the availability of donor organs. Consent legislation is one strategy, among many, for improving donor rates. Other strategies need to be considered in order to alleviate the organ donor shortage. For example, donor rates may be improved by introducing aspects of the Spanish Model, increasing the transplant capacity (for example, more trained surgeons and transplant centers, and improving the ability to identify potential donors. Also, given the relatively low levels of living donation in opt-out systems, it may be possible to reduce the number of people on waiting lists by developing these countries’ living organ donation infrastructure and by presenting this option to relatives. Although living organ donation is only used to supplement deceased donation, presenting this option may save the lives of patients who are unlikely to receive an organ from a deceased donor. Indeed, research in Spain has found that the introduction of living organ donation information programs can increase uptake of this type of donation [43]. The use of such programs has the potential to increase living donation, which is likely to help alleviate the shortage of donor organs [44].

### Endnotes

^1^We used a time invariant measure of GDP to make sure that variation between countries’ GDP was not responsible for the effect of consent legislation, allowing us to compare the relative wealth of countries on average rather than financial growth (change in GDP) that is influenced by many internal and external factors.

^2^Using data from previous research [38], we compared whether the number of critical care beds varies between opt-in and opt-out countries. This previous research only had critical care bed data for 23 (7 opt-in and 16 opt-out) of the countries in our sample. We did not include this data in the analysis because the sample size was too small. However, we analyzed the data from these 23 countries in order to assess whether there were any differences between opt-in and opt-out countries. Because there was only one year of data (2010) an analysis of variance (ANOVA) was used to assess the results rather than MLM. The initial ANOVA simply assessed the effect of consent on the number of critical care beds per 100,000 people in the population, without the inclusion of any covariates. This analysis found that although there were more critical care beds in opt-in (*M* = 12.90, *SD* = 9.09) than opt-out countries (*M* = 10.91, *SD* = 4.67), this difference was not significant (*P* = .49). Next, we repeated the analysis controlling for GDP, RTA, number of hospital beds, Catholicism, and legal system. The value of these Level 2 variables was the same as those used in the above models. Importantly, consent remained a non-significant predictor of critical care beds after controlling for these covariates (*P* = .13). We also found that the number of critical care beds was strongly correlated with the number of hospital beds (*r* = .66, *P* = .001). This suggests that by including the number of hospital beds into the MLMs in the main analyses we are partly accounting for the number of critical care beds.

## Abbreviations

CI: Confidence intervals
DBD: donation after brain stem death
DCD: donation after cardiac death
EC2SLS: error component two-stage least squares
GDP: gross domestic product
GLS: generalized least squares
IRODaT: International Registry of Organ Donation
IV: instrumental variable
pmp: per million population
RTA: road traffic accident
SD: standard deviation
WGI: World Giving Index
